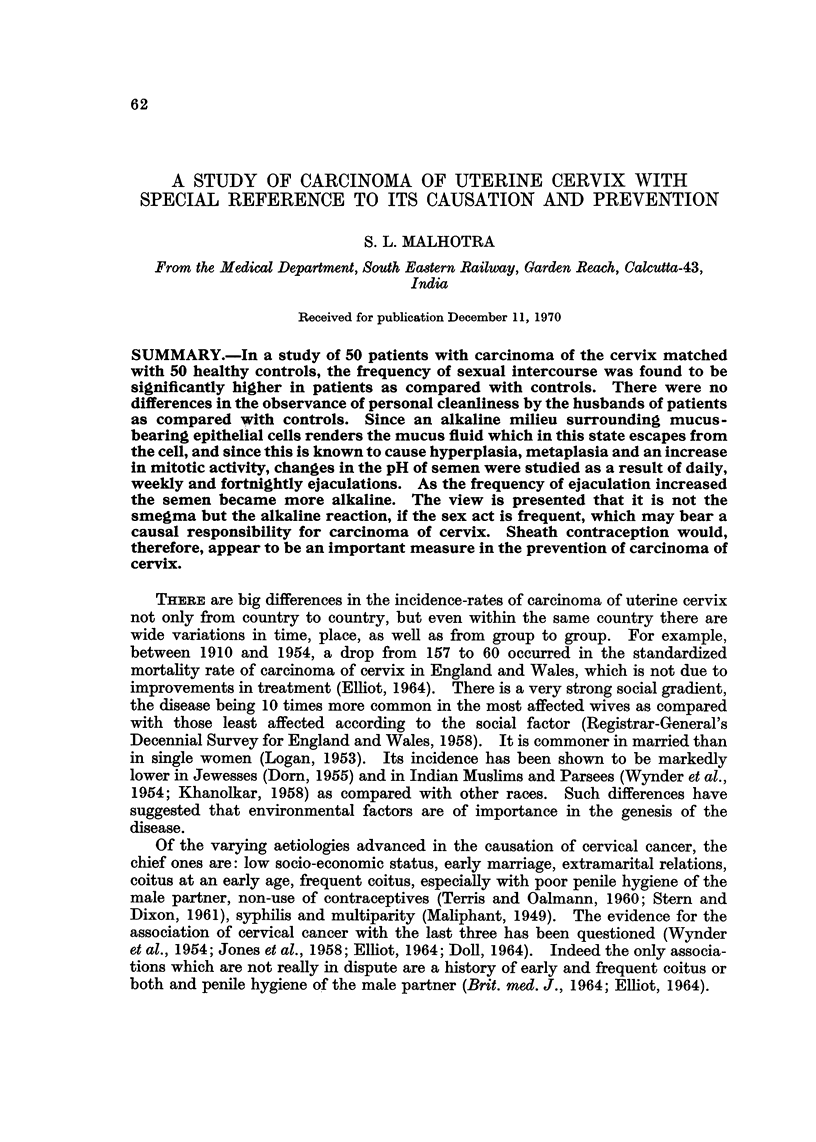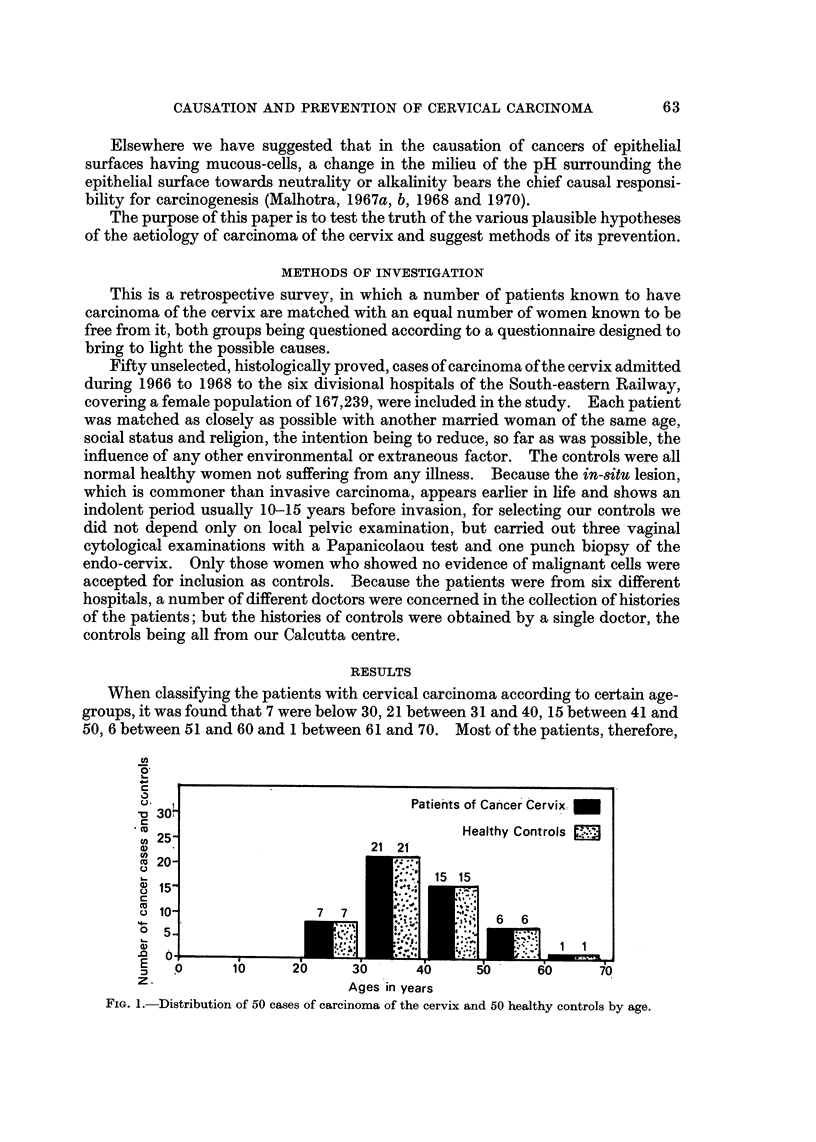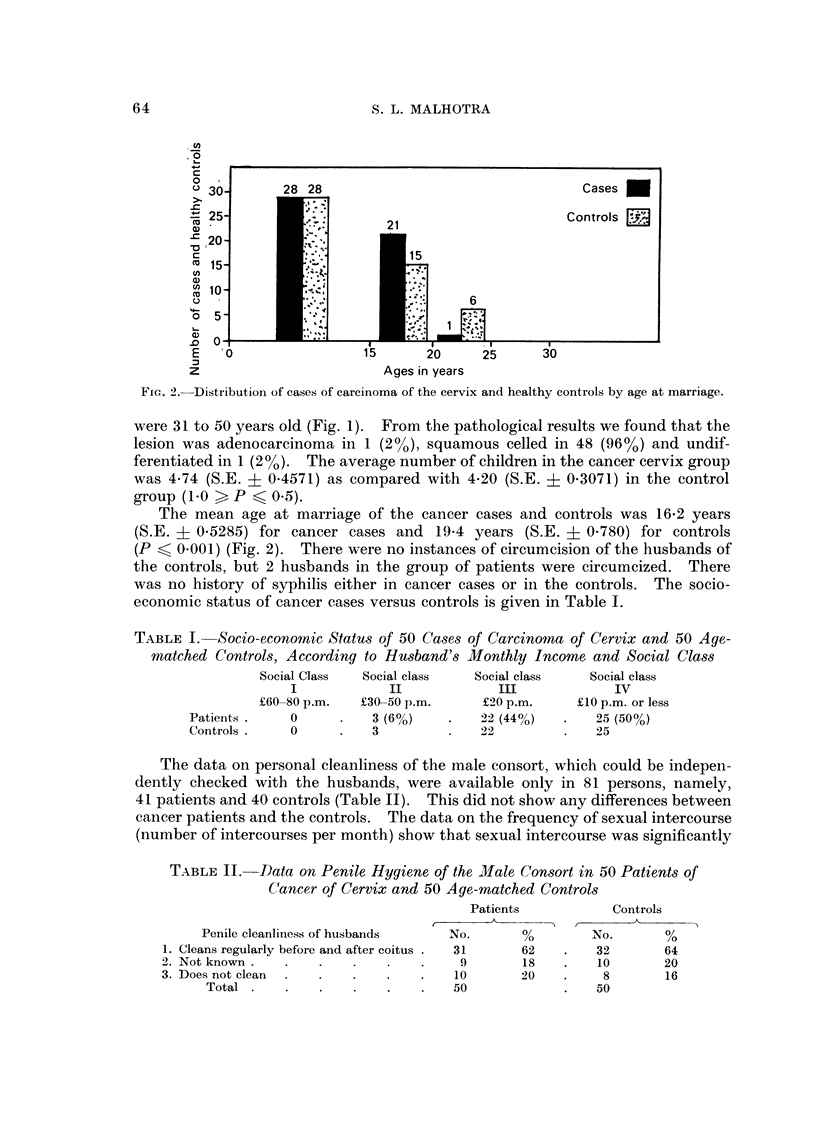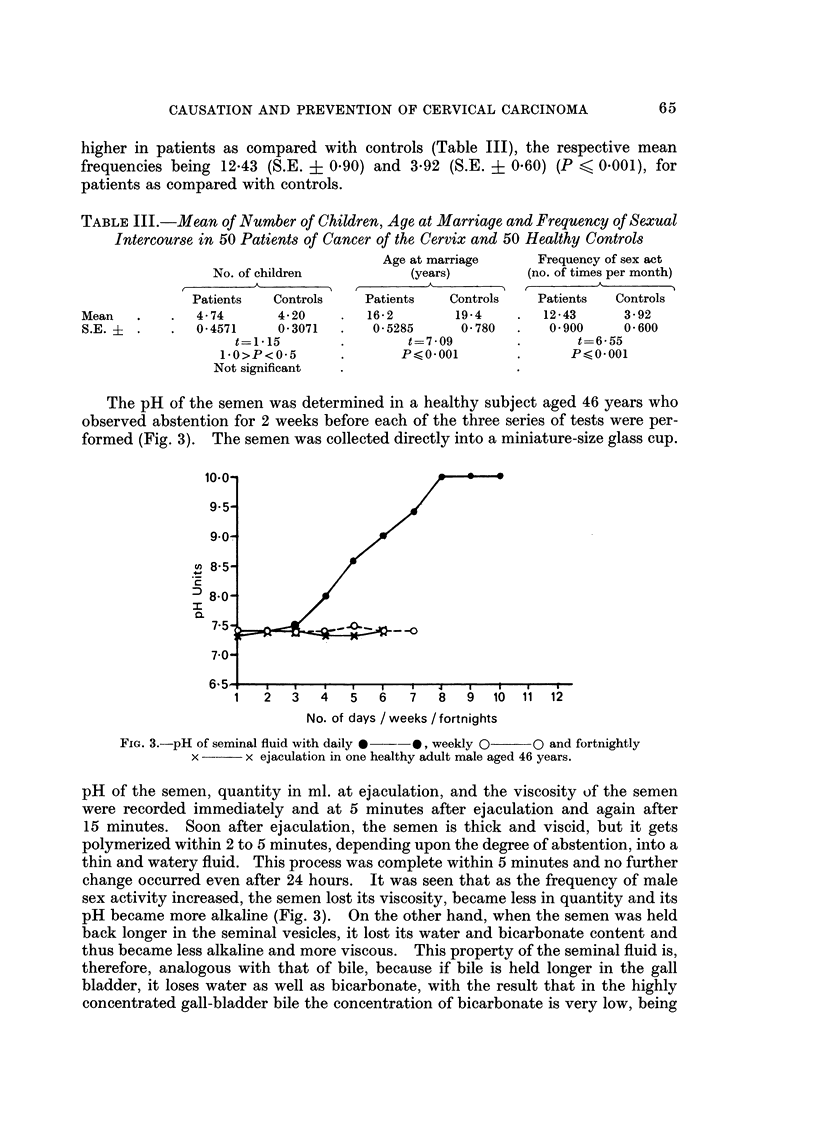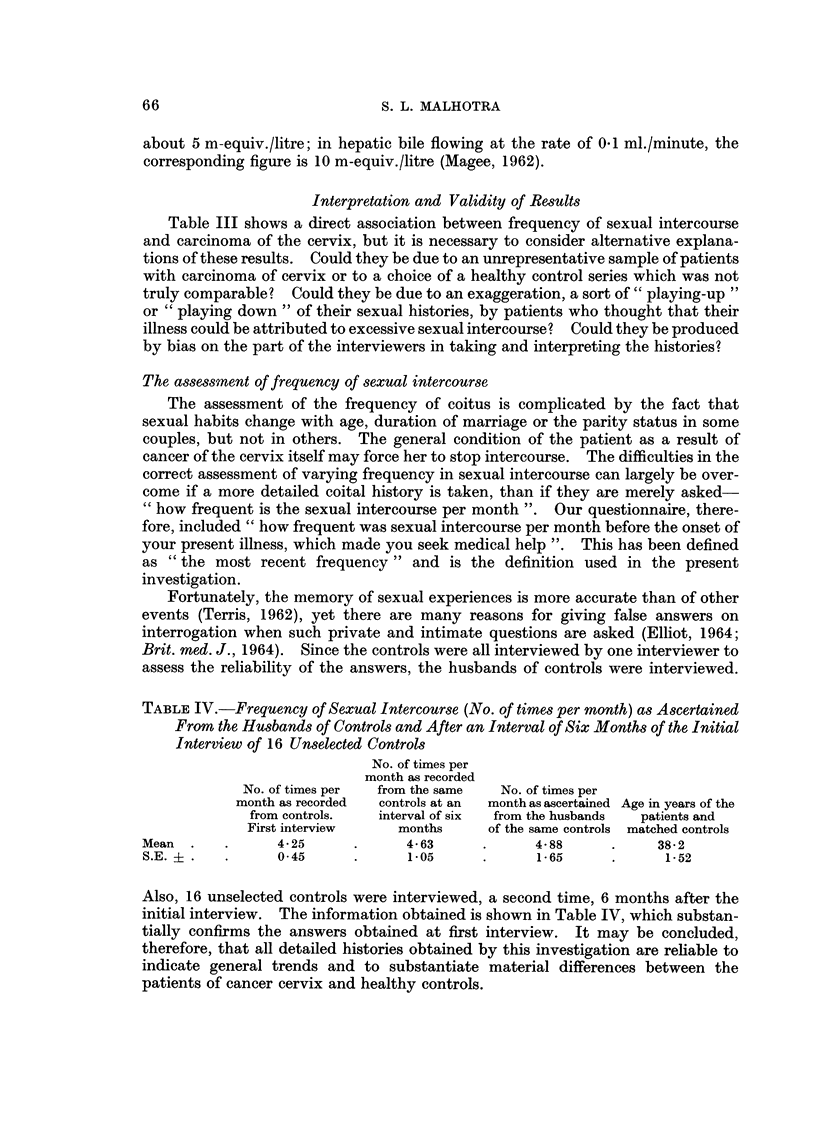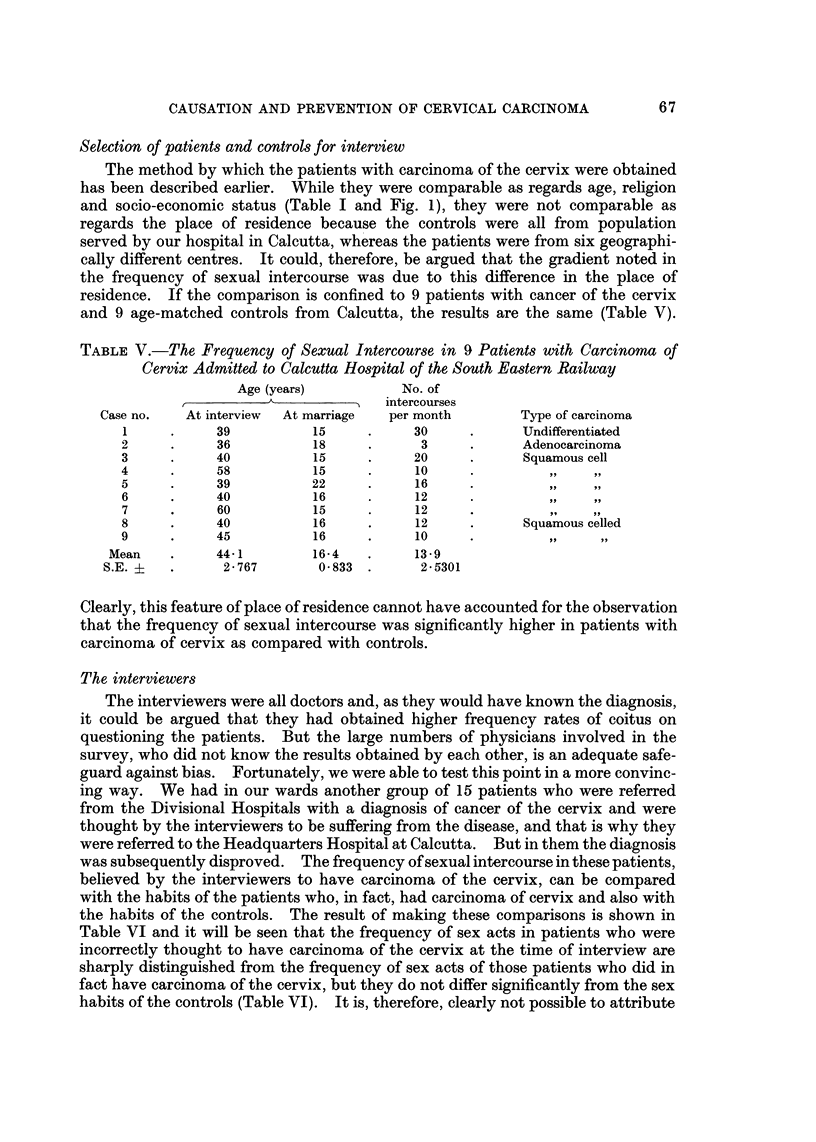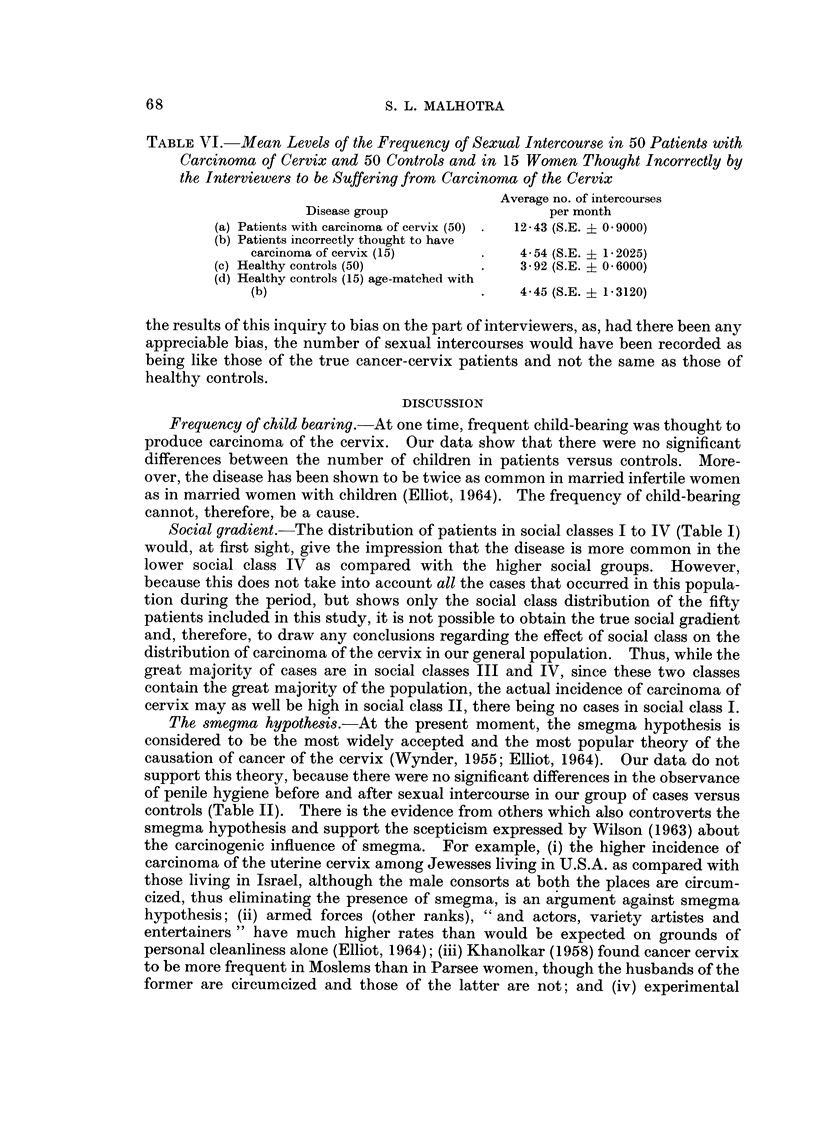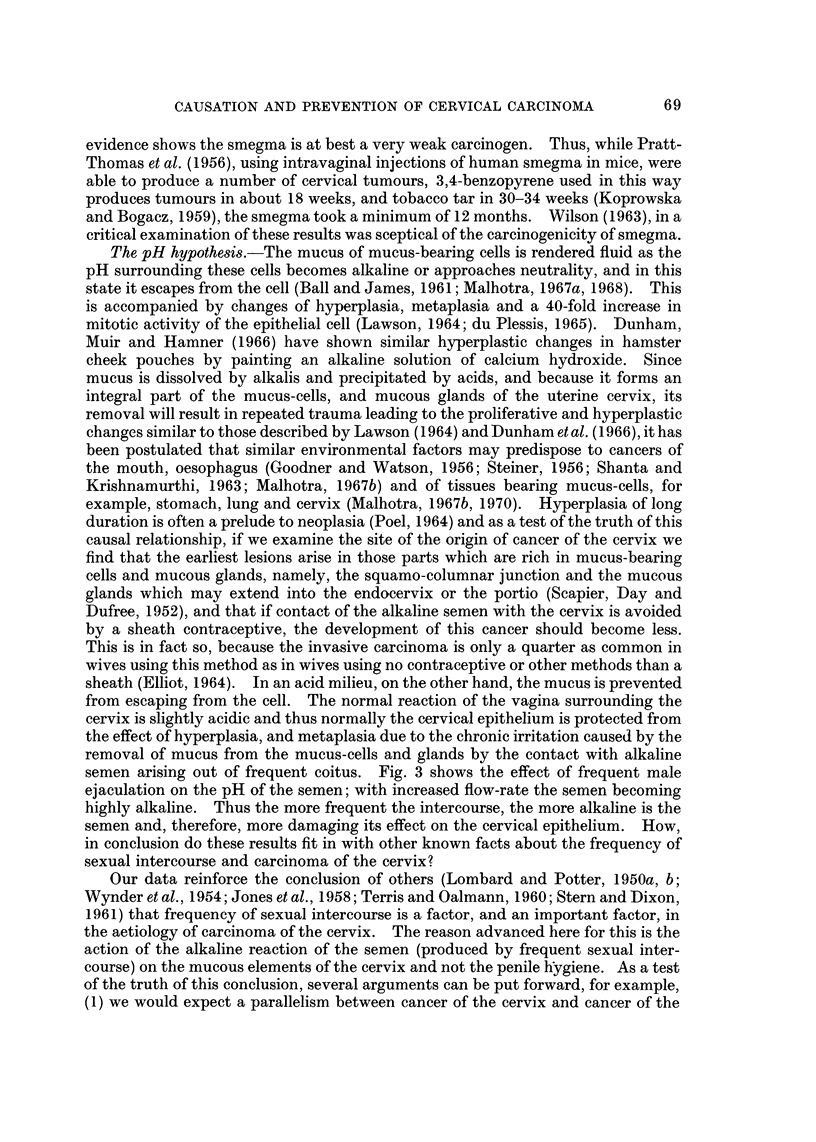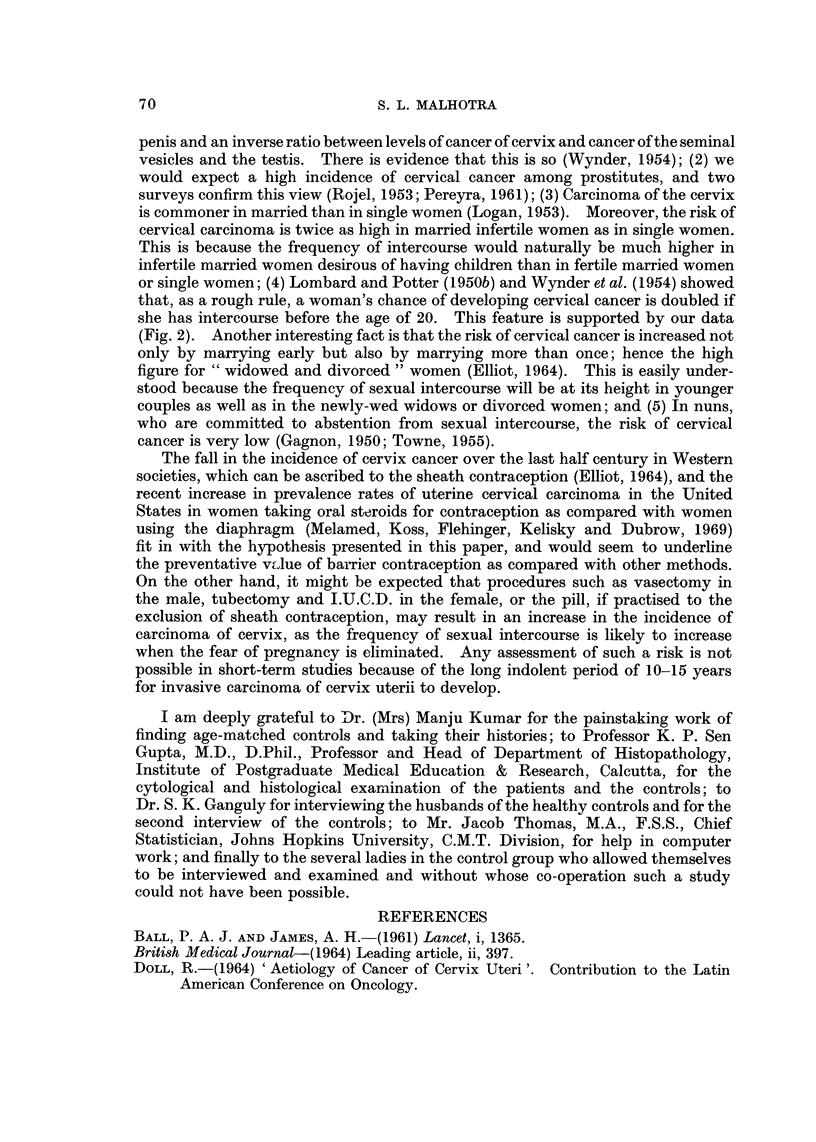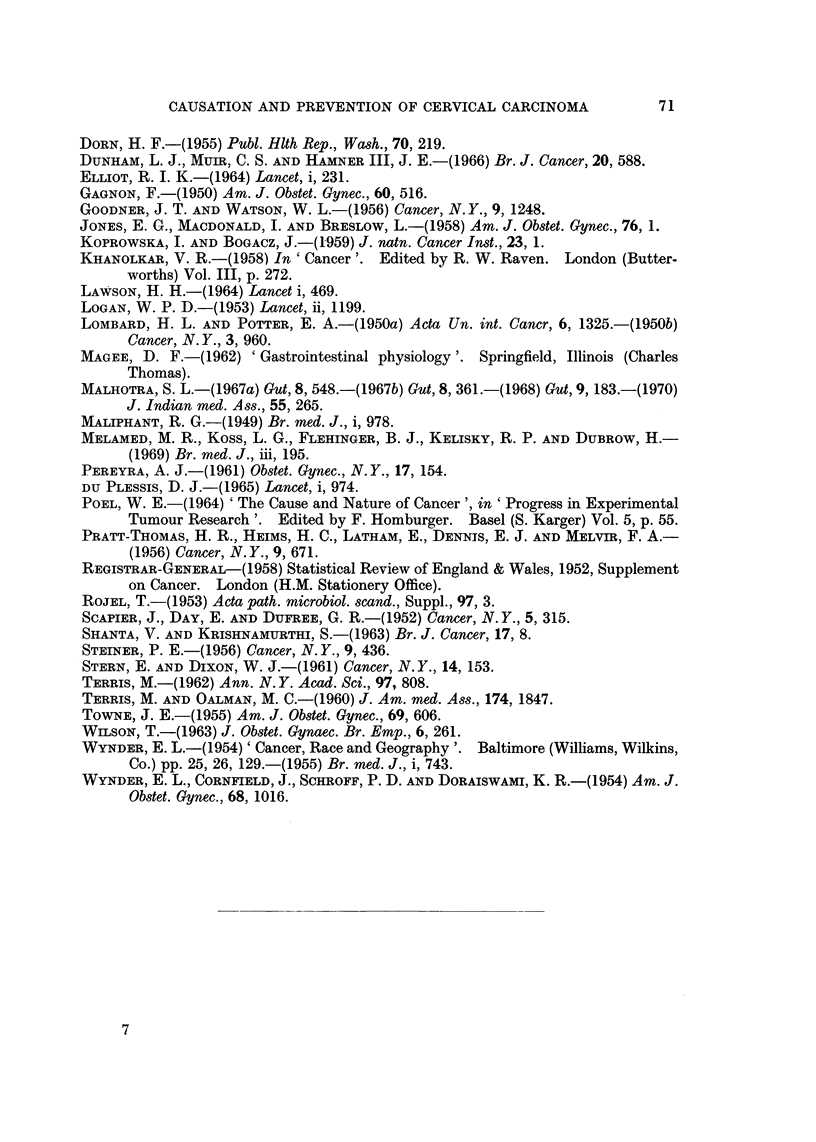# A Study of Carcinoma of Uterine Cervix with Special Reference to its Causation and Prevention

**DOI:** 10.1038/bjc.1971.9

**Published:** 1971-03

**Authors:** S. L. Malhotra

## Abstract

In a study of 50 patients with carcinoma of the cervix matched with 50 healthy controls, the frequency of sexual intercourse was found to be significantly higher in patients as compared with controls. There were no differences in the observance of personal cleanliness by the husbands of patients as compared with controls. Since an alkaline milieu surrounding mucus-bearing epithelial cells renders the mucus fluid which in this state escapes from the cell, and since this is known to cause hyperplasia, metaplasia and an increase in mitotic activity, changes in the pH of semen were studied as a result of daily, weekly and fortnightly ejaculations. As the frequency of ejaculation increased the semen became more alkaline. The view is presented that it is not the smegma but the alkaline reaction, if the sex act is frequent, which may bear a causal responsibility for carcinoma of cervix. Sheath contraception would, therefore, appear to be an important measure in the prevention of carcinoma of cervix.


### A STUDY OF CARCINOMA OF UTERINE CERVIX WITH SPECIAL REFERENCE TO ITS CAUSATION AND PREVENTION

S. L. MALHOTRA

*From the Medical Department, South Eastern Railway, Garden Reach, Calcutta-43, India*

Received for publication December 11, 1970

**SUMMARY.—In a study of 50 patients with carcinoma of the cervix matched with 50 healthy controls, the frequency of sexual intercourse was found to be significantly higher in patients as compared with controls. There were no differences in the observance of personal cleanliness by the husbands of patients as compared with controls. Since an alkaline milieu surrounding mucus-bearing epithelial cells renders the mucus fluid which in this state escapes from the cell, and since this is known to cause hyperplasia, metaplasia and an increase in mitotic activity, changes in the pH of semen were studied as a result of daily, weekly and fortnightly ejaculations. As the frequency of ejaculation increased the semen became more alkaline. The view is presented that it is not the smegma but the alkaline reaction, if the sex act is frequent, which may bear a causal responsibility for carcinoma of cervix. Sheath contraception would, therefore, appear to be an important measure in the prevention of carcinoma of cervix.**

THERE are big differences in the incidence-rates of carcinoma of uterine cervix not only from country to country, but even within the same country there are wide variations in time, place, as well as from group to group. For example, between 1910 and 1954, a drop from 157 to 60 occurred in the standardized mortality rate of carcinoma of cervix in England and Wales, which is not due to improvements in treatment (Elliot, 1964). There is a very strong social gradient, the disease being 10 times more common in the most affected wives as compared with those least affected according to the social factor (Registrar-General's Decennial Survey for England and Wales, 1958). It is commoner in married than in single women (Logan, 1953). Its incidence has been shown to be markedly lower in Jewesses (Dorn, 1955) and in Indian Muslims and Parsees (Wynder *et al.*, 1954; Khanolkar, 1958) as compared with other races. Such differences have suggested that environmental factors are of importance in the genesis of the disease.

Of the varying aetiologies advanced in the causation of cervical cancer, the chief ones are: low socio-economic status, early marriage, extramarital relations, coitus at an early age, frequent coitus, especially with poor penile hygiene of the male partner, non-use of contraceptives (Terris and Oalmann, 1960; Stern and Dixon, 1961), syphilis and multiparity (Maliphant, 1949). The evidence for the association of cervical cancer with the last three has been questioned (Wynder *et al.*, 1954; Jones *et al.*, 1958; Elliot, 1964; Doll, 1964). Indeed the only associations which are not really in dispute are a history of early and frequent coitus or both and penile hygiene of the male partner (*Brit. med. J.*, 1964; Elliot, 1964).

Elsewhere we have suggested that in the causation of cancers of epithelial surfaces having mucous-cells, a change in the milieu of the pH surrounding the epithelial surface towards neutrality or alkalinity bears the chief causal responsibility for carcinogenesis (Malhotra, 1967*a*, *b*, 1968 and 1970).

The purpose of this paper is to test the truth of the various plausible hypotheses of the aetiology of carcinoma of the cervix and suggest methods of its prevention.

### METHODS OF INVESTIGATION

This is a retrospective survey, in which a number of patients known to have carcinoma of the cervix are matched with an equal number of women known to be free from it, both groups being questioned according to a questionnaire designed to bring to light the possible causes.

Fifty unselected, histologically proved, cases of carcinoma of the cervix admitted during 1966 to 1968 to the six divisional hospitals of the South-eastern Railway, covering a female population of 167,239, were included in the study. Each patient was matched as closely as possible with another married woman of the same age, social status and religion, the intention being to reduce, so far as was possible, the influence of any other environmental or extraneous factor. The controls were all normal healthy women not suffering from any illness. Because the *in-situ* lesion, which is commoner than invasive carcinoma, appears earlier in life and shows an indolent period usually 10–15 years before invasion, for selecting our controls we did not depend only on local pelvic examination, but carried out three vaginal cytological examinations with a Papanicolaou test and one punch biopsy of the endo-cervix. Only those women who showed no evidence of malignant cells were accepted for inclusion as controls. Because the patients were from six different hospitals, a number of different doctors were concerned in the collection of histories of the patients; but the histories of controls were obtained by a single doctor, the controls being all from our Calcutta centre.

### RESULTS

When classifying the patients with cervical carcinoma according to certain age-groups, it was found that 7 were below 30, 21 between 31 and 40, 15 between 41 and 50, 6 between 51 and 60 and 1 between 61 and 70. Most of the patients, therefore,

FIG. 1.—Distribution of 50 cases of carcinoma of the cervix and 50 healthy controls by age.

FIG. 2.—Distribution of cases of carcinoma of the cervix and healthy controls by age at marriage.

were 31 to 50 years old (Fig. 1). From the pathological results we found that the lesion was adenocarcinoma in 1 (2%), squamous celled in 48 (96%) and undifferentiated in 1 (2%). The average number of children in the cancer cervix group was 4·74 (S.E. $\pm$ 0·4571) as compared with 4·20 (S.E. $\pm$ 0·3071) in the control group ($1{\cdot}0 \geqslant P \leqslant 0{\cdot}5$).

The mean age at marriage of the cancer cases and controls was 16·2 years (S.E. $\pm$ 0·5285) for cancer cases and 19·4 years (S.E. $\pm$ 0·780) for controls ($P \leqslant 0{\cdot}001$) (Fig. 2). There were no instances of circumcision of the husbands of the controls, but 2 husbands in the group of patients were circumcized. There was no history of syphilis either in cancer cases or in the controls. The socio-economic status of cancer cases versus controls is given in Table I.

TABLE I.—*Socio-economic Status of* 50 *Cases of Carcinoma of Cervix and* 50 *Age-matched Controls, According to Husband's Monthly Income and Social Class*

| | Social Class I £60–80 p.m. | Social class II £30–50 p.m. | Social class III £20 p.m. | Social class IV £10 p.m. or less |
|---|---|---|---|---|
| Patients | 0 | 3 (6%) | 22 (44%) | 25 (50%) |
| Controls | 0 | 3 | 22 | 25 |

The data on personal cleanliness of the male consort, which could be independently checked with the husbands, were available only in 81 persons, namely, 41 patients and 40 controls (Table II). This did not show any differences between cancer patients and the controls. The data on the frequency of sexual intercourse (number of intercourses per month) show that sexual intercourse was significantly

TABLE II.—*Data on Penile Hygiene of the Male Consort in* 50 *Patients of Cancer of Cervix and* 50 *Age-matched Controls*

| | Patients | | Controls | |
|---|---|---|---|---|
| Penile cleanliness of husbands | No. | % | No. | % |
| 1. Cleans regularly before and after coitus | 31 | 62 | 32 | 64 |
| 2. Not known | 9 | 18 | 10 | 20 |
| 3. Does not clean | 10 | 20 | 8 | 16 |
| Total | 50 | | 50 | |

higher in patients as compared with controls (Table III), the respective mean frequencies being 12·43 (S.E. ± 0·90) and 3·92 (S.E. ± 0·60) ($P \leqslant 0{\cdot}001$), for patients as compared with controls.

TABLE III.—*Mean of Number of Children, Age at Marriage and Frequency of Sexual Intercourse in 50 Patients of Cancer of the Cervix and 50 Healthy Controls*

| | No. of children | | Age at marriage (years) | | Frequency of sex act (no. of times per month) | |
|---|---|---|---|---|---|---|
| | Patients | Controls | Patients | Controls | Patients | Controls |
| Mean | 4·74 | 4·20 | 16·2 | 19·4 | 12·43 | 3·92 |
| S.E. ± | 0·4571 | 0·3071 | 0·5285 | 0·780 | 0·900 | 0·600 |
| | $t=1{\cdot}15$ | | $t=7{\cdot}09$ | | $t=6{\cdot}55$ | |
| | $1{\cdot}0>P<0{\cdot}5$ | | $P\leqslant 0{\cdot}001$ | | $P\leqslant 0{\cdot}001$ | |
| | Not significant | | | | | |

The pH of the semen was determined in a healthy subject aged 46 years who observed abstention for 2 weeks before each of the three series of tests were performed (Fig. 3). The semen was collected directly into a miniature-size glass cup.

FIG. 3.—pH of seminal fluid with daily ●———●, weekly ○———○ and fortnightly ×———× ejaculation in one healthy adult male aged 46 years.

pH of the semen, quantity in ml. at ejaculation, and the viscosity of the semen were recorded immediately and at 5 minutes after ejaculation and again after 15 minutes. Soon after ejaculation, the semen is thick and viscid, but it gets polymerized within 2 to 5 minutes, depending upon the degree of abstention, into a thin and watery fluid. This process was complete within 5 minutes and no further change occurred even after 24 hours. It was seen that as the frequency of male sex activity increased, the semen lost its viscosity, became less in quantity and its pH became more alkaline (Fig. 3). On the other hand, when the semen was held back longer in the seminal vesicles, it lost its water and bicarbonate content and thus became less alkaline and more viscous. This property of the seminal fluid is, therefore, analogous with that of bile, because if bile is held longer in the gall bladder, it loses water as well as bicarbonate, with the result that in the highly concentrated gall-bladder bile the concentration of bicarbonate is very low, being about 5 m-equiv./litre; in hepatic bile flowing at the rate of 0·1 ml./minute, the corresponding figure is 10 m-equiv./litre (Magee, 1962).

### *Interpretation and Validity of Results*

Table III shows a direct association between frequency of sexual intercourse and carcinoma of the cervix, but it is necessary to consider alternative explanations of these results. Could they be due to an unrepresentative sample of patients with carcinoma of cervix or to a choice of a healthy control series which was not truly comparable? Could they be due to an exaggeration, a sort of " playing-up " or " playing down " of their sexual histories, by patients who thought that their illness could be attributed to excessive sexual intercourse? Could they be produced by bias on the part of the interviewers in taking and interpreting the histories?

*The assessment of frequency of sexual intercourse*

The assessment of the frequency of coitus is complicated by the fact that sexual habits change with age, duration of marriage or the parity status in some couples, but not in others. The general condition of the patient as a result of cancer of the cervix itself may force her to stop intercourse. The difficulties in the correct assessment of varying frequency in sexual intercourse can largely be overcome if a more detailed coital history is taken, than if they are merely asked—" how frequent is the sexual intercourse per month ". Our questionnaire, therefore, included " how frequent was sexual intercourse per month before the onset of your present illness, which made you seek medical help ". This has been defined as " the most recent frequency " and is the definition used in the present investigation.

Fortunately, the memory of sexual experiences is more accurate than of other events (Terris, 1962), yet there are many reasons for giving false answers on interrogation when such private and intimate questions are asked (Elliot, 1964; *Brit. med. J.*, 1964). Since the controls were all interviewed by one interviewer to assess the reliability of the answers, the husbands of controls were interviewed.

TABLE IV.—*Frequency of Sexual Intercourse (No. of times per month) as Ascertained From the Husbands of Controls and After an Interval of Six Months of the Initial Interview of* 16 *Unselected Controls*

| | No. of times per month as recorded from controls. First interview | No. of times per month as recorded from the same controls at an interval of six months | No. of times per month as ascertained from the husbands of the same controls | Age in years of the patients and matched controls |
|---|---|---|---|---|
| Mean | 4·25 | 4·63 | 4·88 | 38·2 |
| S.E. ± | 0·45 | 1·05 | 1·65 | 1·52 |

Also, 16 unselected controls were interviewed, a second time, 6 months after the initial interview. The information obtained is shown in Table IV, which substantially confirms the answers obtained at first interview. It may be concluded, therefore, that all detailed histories obtained by this investigation are reliable to indicate general trends and to substantiate material differences between the patients of cancer cervix and healthy controls.

*Selection of patients and controls for interview*

The method by which the patients with carcinoma of the cervix were obtained has been described earlier. While they were comparable as regards age, religion and socio-economic status (Table I and Fig. 1), they were not comparable as regards the place of residence because the controls were all from population served by our hospital in Calcutta, whereas the patients were from six geographically different centres. It could, therefore, be argued that the gradient noted in the frequency of sexual intercourse was due to this difference in the place of residence. If the comparison is confined to 9 patients with cancer of the cervix and 9 age-matched controls from Calcutta, the results are the same (Table V).

TABLE V.—*The Frequency of Sexual Intercourse in 9 Patients with Carcinoma of Cervix Admitted to Calcutta Hospital of the South Eastern Railway*

| Case no. | Age (years) At interview | Age (years) At marriage | No. of intercourses per month | Type of carcinoma |
|---|---|---|---|---|
| 1 | 39 | 15 | 30 | Undifferentiated |
| 2 | 36 | 18 | 3 | Adenocarcinoma |
| 3 | 40 | 15 | 20 | Squamous cell |
| 4 | 58 | 15 | 10 | ,, ,, |
| 5 | 39 | 22 | 16 | ,, ,, |
| 6 | 40 | 16 | 12 | ,, ,, |
| 7 | 60 | 15 | 12 | ,, ,, |
| 8 | 40 | 16 | 12 | Squamous celled |
| 9 | 45 | 16 | 10 | ,, ,, |
| Mean | 44·1 | 16·4 | 13·9 | |
| S.E. ± | 2·767 | 0·833 | 2·5301 | |

Clearly, this feature of place of residence cannot have accounted for the observation that the frequency of sexual intercourse was significantly higher in patients with carcinoma of cervix as compared with controls.

*The interviewers*

The interviewers were all doctors and, as they would have known the diagnosis, it could be argued that they had obtained higher frequency rates of coitus on questioning the patients. But the large numbers of physicians involved in the survey, who did not know the results obtained by each other, is an adequate safeguard against bias. Fortunately, we were able to test this point in a more convincing way. We had in our wards another group of 15 patients who were referred from the Divisional Hospitals with a diagnosis of cancer of the cervix and were thought by the interviewers to be suffering from the disease, and that is why they were referred to the Headquarters Hospital at Calcutta. But in them the diagnosis was subsequently disproved. The frequency of sexual intercourse in these patients, believed by the interviewers to have carcinoma of the cervix, can be compared with the habits of the patients who, in fact, had carcinoma of cervix and also with the habits of the controls. The result of making these comparisons is shown in Table VI and it will be seen that the frequency of sex acts in patients who were incorrectly thought to have carcinoma of the cervix at the time of interview are sharply distinguished from the frequency of sex acts of those patients who did in fact have carcinoma of the cervix, but they do not differ significantly from the sex habits of the controls (Table VI). It is, therefore, clearly not possible to attribute

TABLE VI.—*Mean Levels of the Frequency of Sexual Intercourse in 50 Patients with Carcinoma of Cervix and 50 Controls and in 15 Women Thought Incorrectly by the Interviewers to be Suffering from Carcinoma of the Cervix*

| Disease group | Average no. of intercourses per month |
|---|---|
| (a) Patients with carcinoma of cervix (50) . | 12·43 (S.E. ± 0·9000) |
| (b) Patients incorrectly thought to have carcinoma of cervix (15) . | 4·54 (S.E. ± 1·2025) |
| (c) Healthy controls (50) . | 3·92 (S.E. ± 0·6000) |
| (d) Healthy controls (15) age-matched with (b) . | 4·45 (S.E. ± 1·3120) |

the results of this inquiry to bias on the part of interviewers, as, had there been any appreciable bias, the number of sexual intercourses would have been recorded as being like those of the true cancer-cervix patients and not the same as those of healthy controls.

### DISCUSSION

*Frequency of child bearing.*—At one time, frequent child-bearing was thought to produce carcinoma of the cervix. Our data show that there were no significant differences between the number of children in patients versus controls. Moreover, the disease has been shown to be twice as common in married infertile women as in married women with children (Elliot, 1964). The frequency of child-bearing cannot, therefore, be a cause.

*Social gradient.*—The distribution of patients in social classes I to IV (Table I) would, at first sight, give the impression that the disease is more common in the lower social class IV as compared with the higher social groups. However, because this does not take into account *all* the cases that occurred in this population during the period, but shows only the social class distribution of the fifty patients included in this study, it is not possible to obtain the true social gradient and, therefore, to draw any conclusions regarding the effect of social class on the distribution of carcinoma of the cervix in our general population. Thus, while the great majority of cases are in social classes III and IV, since these two classes contain the great majority of the population, the actual incidence of carcinoma of cervix may as well be high in social class II, there being no cases in social class I.

*The smegma hypothesis.*—At the present moment, the smegma hypothesis is considered to be the most widely accepted and the most popular theory of the causation of cancer of the cervix (Wynder, 1955; Elliot, 1964). Our data do not support this theory, because there were no significant differences in the observance of penile hygiene before and after sexual intercourse in our group of cases versus controls (Table II). There is the evidence from others which also controverts the smegma hypothesis and support the scepticism expressed by Wilson (1963) about the carcinogenic influence of smegma. For example, (i) the higher incidence of carcinoma of the uterine cervix among Jewesses living in U.S.A. as compared with those living in Israel, although the male consorts at both the places are circumcized, thus eliminating the presence of smegma, is an argument against smegma hypothesis; (ii) armed forces (other ranks), " and actors, variety artistes and entertainers " have much higher rates than would be expected on grounds of personal cleanliness alone (Elliot, 1964); (iii) Khanolkar (1958) found cancer cervix to be more frequent in Moslems than in Parsee women, though the husbands of the former are circumcized and those of the latter are not; and (iv) experimental evidence shows the smegma is at best a very weak carcinogen. Thus, while Pratt-Thomas *et al.* (1956), using intravaginal injections of human smegma in mice, were able to produce a number of cervical tumours, 3,4-benzopyrene used in this way produces tumours in about 18 weeks, and tobacco tar in 30–34 weeks (Koprowska and Bogacz, 1959), the smegma took a minimum of 12 months. Wilson (1963), in a critical examination of these results was sceptical of the carcinogenicity of smegma.

*The pH hypothesis.*—The mucus of mucus-bearing cells is rendered fluid as the pH surrounding these cells becomes alkaline or approaches neutrality, and in this state it escapes from the cell (Ball and James, 1961; Malhotra, 1967*a*, 1968). This is accompanied by changes of hyperplasia, metaplasia and a 40-fold increase in mitotic activity of the epithelial cell (Lawson, 1964; du Plessis, 1965). Dunham, Muir and Hamner (1966) have shown similar hyperplastic changes in hamster cheek pouches by painting an alkaline solution of calcium hydroxide. Since mucus is dissolved by alkalis and precipitated by acids, and because it forms an integral part of the mucus-cells, and mucous glands of the uterine cervix, its removal will result in repeated trauma leading to the proliferative and hyperplastic changes similar to those described by Lawson (1964) and Dunham *et al.* (1966), it has been postulated that similar environmental factors may predispose to cancers of the mouth, oesophagus (Goodner and Watson, 1956; Steiner, 1956; Shanta and Krishnamurthi, 1963; Malhotra, 1967*b*) and of tissues bearing mucus-cells, for example, stomach, lung and cervix (Malhotra, 1967*b*, 1970). Hyperplasia of long duration is often a prelude to neoplasia (Poel, 1964) and as a test of the truth of this causal relationship, if we examine the site of the origin of cancer of the cervix we find that the earliest lesions arise in those parts which are rich in mucus-bearing cells and mucous glands, namely, the squamo-columnar junction and the mucous glands which may extend into the endocervix or the portio (Scapier, Day and Dufree, 1952), and that if contact of the alkaline semen with the cervix is avoided by a sheath contraceptive, the development of this cancer should become less. This is in fact so, because the invasive carcinoma is only a quarter as common in wives using this method as in wives using no contraceptive or other methods than a sheath (Elliot, 1964). In an acid milieu, on the other hand, the mucus is prevented from escaping from the cell. The normal reaction of the vagina surrounding the cervix is slightly acidic and thus normally the cervical epithelium is protected from the effect of hyperplasia, and metaplasia due to the chronic irritation caused by the removal of mucus from the mucus-cells and glands by the contact with alkaline semen arising out of frequent coitus. Fig. 3 shows the effect of frequent male ejaculation on the pH of the semen; with increased flow-rate the semen becoming highly alkaline. Thus the more frequent the intercourse, the more alkaline is the semen and, therefore, more damaging its effect on the cervical epithelium. How, in conclusion do these results fit in with other known facts about the frequency of sexual intercourse and carcinoma of the cervix?

Our data reinforce the conclusion of others (Lombard and Potter, 1950*a*, *b*; Wynder *et al.*, 1954; Jones *et al.*, 1958; Terris and Oalmann, 1960; Stern and Dixon, 1961) that frequency of sexual intercourse is a factor, and an important factor, in the aetiology of carcinoma of the cervix. The reason advanced here for this is the action of the alkaline reaction of the semen (produced by frequent sexual intercourse) on the mucous elements of the cervix and not the penile hygiene. As a test of the truth of this conclusion, several arguments can be put forward, for example, (1) we would expect a parallelism between cancer of the cervix and cancer of the penis and an inverse ratio between levels of cancer of cervix and cancer of the seminal vesicles and the testis. There is evidence that this is so (Wynder, 1954); (2) we would expect a high incidence of cervical cancer among prostitutes, and two surveys confirm this view (Rojel, 1953; Pereyra, 1961); (3) Carcinoma of the cervix is commoner in married than in single women (Logan, 1953). Moreover, the risk of cervical carcinoma is twice as high in married infertile women as in single women. This is because the frequency of intercourse would naturally be much higher in infertile married women desirous of having children than in fertile married women or single women; (4) Lombard and Potter (1950*b*) and Wynder *et al.* (1954) showed that, as a rough rule, a woman's chance of developing cervical cancer is doubled if she has intercourse before the age of 20. This feature is supported by our data (Fig. 2). Another interesting fact is that the risk of cervical cancer is increased not only by marrying early but also by marrying more than once; hence the high figure for " widowed and divorced " women (Elliot, 1964). This is easily understood because the frequency of sexual intercourse will be at its height in younger couples as well as in the newly-wed widows or divorced women; and (5) In nuns, who are committed to abstention from sexual intercourse, the risk of cervical cancer is very low (Gagnon, 1950; Towne, 1955).

The fall in the incidence of cervix cancer over the last half century in Western societies, which can be ascribed to the sheath contraception (Elliot, 1964), and the recent increase in prevalence rates of uterine cervical carcinoma in the United States in women taking oral steroids for contraception as compared with women using the diaphragm (Melamed, Koss, Flehinger, Kelisky and Dubrow, 1969) fit in with the hypothesis presented in this paper, and would seem to underline the preventative value of barrier contraception as compared with other methods. On the other hand, it might be expected that procedures such as vasectomy in the male, tubectomy and I.U.C.D. in the female, or the pill, if practised to the exclusion of sheath contraception, may result in an increase in the incidence of carcinoma of cervix, as the frequency of sexual intercourse is likely to increase when the fear of pregnancy is eliminated. Any assessment of such a risk is not possible in short-term studies because of the long indolent period of 10–15 years for invasive carcinoma of cervix uterii to develop.

I am deeply grateful to Dr. (Mrs) Manju Kumar for the painstaking work of finding age-matched controls and taking their histories; to Professor K. P. Sen Gupta, M.D., D.Phil., Professor and Head of Department of Histopathology, Institute of Postgraduate Medical Education & Research, Calcutta, for the cytological and histological examination of the patients and the controls; to Dr. S. K. Ganguly for interviewing the husbands of the healthy controls and for the second interview of the controls; to Mr. Jacob Thomas, M.A., F.S.S., Chief Statistician, Johns Hopkins University, C.M.T. Division, for help in computer work; and finally to the several ladies in the control group who allowed themselves to be interviewed and examined and without whose co-operation such a study could not have been possible.

### REFERENCES

Ball, P. A. J. and James, A. H.—(1961) *Lancet*, i, 1365.
*British Medical Journal*—(1964) Leading article, ii, 397.
Doll, R.—(1964) ' Aetiology of Cancer of Cervix Uteri '. Contribution to the Latin American Conference on Oncology.

DORN, H. F.—(1955) *Publ. Hlth Rep., Wash.*, **70**, 219.
DUNHAM, L. J., MUIR, C. S. AND HAMNER III, J. E.—(1966) *Br. J. Cancer*, **20**, 588.
ELLIOT, R. I. K.—(1964) *Lancet*, i, 231.
GAGNON, F.—(1950) *Am. J. Obstet. Gynec.*, **60**, 516.
GOODNER, J. T. AND WATSON, W. L.—(1956) *Cancer, N.Y.*, **9**, 1248.
JONES, E. G., MACDONALD, I. AND BRESLOW, L.—(1958) *Am. J. Obstet. Gynec.*, **76**, 1.
KOPROWSKA, I. AND BOGACZ, J.—(1959) *J. natn. Cancer Inst.*, **23**, 1.
KHANOLKAR, V. R.—(1958) *In* ' Cancer '. Edited by R. W. Raven. London (Butterworths) Vol. III, p. 272.
LAWSON, H. H.—(1964) *Lancet* i, 469.
LOGAN, W. P. D.—(1953) *Lancet*, ii, 1199.
LOMBARD, H. L. AND POTTER, E. A.—(1950*a*) *Acta Un. int. Cancr*, **6**, 1325.—(1950*b*) *Cancer, N.Y.*, **3**, 960.
MAGEE, D. F.—(1962) ' Gastrointestinal physiology '. Springfield, Illinois (Charles Thomas).
MALHOTRA, S. L.—(1967*a*) *Gut*, **8**, 548.—(1967*b*) *Gut*, **8**, 361.—(1968) *Gut*, **9**, 183.—(1970) *J. Indian med. Ass.*, **55**, 265.
MALIPHANT, R. G.—(1949) *Br. med. J.*, i, 978.
MELAMED, M. R., KOSS, L. G., FLEHINGER, B. J., KELISKY, R. P. AND DUBROW, H.—(1969) *Br. med. J.*, iii, 195.
PEREYRA, A. J.—(1961) *Obstet. Gynec., N.Y.*, **17**, 154.
DU PLESSIS, D. J.—(1965) *Lancet*, i, 974.
POEL, W. E.—(1964) ' The Cause and Nature of Cancer ', *in* ' Progress in Experimental Tumour Research '. Edited by F. Homburger. Basel (S. Karger) Vol. 5, p. 55.
PRATT-THOMAS, H. R., HEIMS, H. C., LATHAM, E., DENNIS, E. J. AND MELVIR, F. A.—(1956) *Cancer, N.Y.*, **9**, 671.
REGISTRAR-GENERAL—(1958) Statistical Review of England & Wales, 1952, Supplement on Cancer. London (H.M. Stationery Office).
ROJEL, T.—(1953) *Acta path. microbiol. scand.*, Suppl., **97**, 3.
SCAPIER, J., DAY, E. AND DUFREE, G. R.—(1952) *Cancer, N.Y.*, **5**, 315.
SHANTA, V. AND KRISHNAMURTHI, S.—(1963) *Br. J. Cancer*, **17**, 8.
STEINER, P. E.—(1956) *Cancer, N.Y.*, **9**, 436.
STERN, E. AND DIXON, W. J.—(1961) *Cancer, N.Y.*, **14**, 153.
TERRIS, M.—(1962) *Ann. N.Y. Acad. Sci.*, **97**, 808.
TERRIS, M. AND OALMAN, M. C.—(1960) *J. Am. med. Ass.*, **174**, 1847.
TOWNE, J. E.—(1955) *Am. J. Obstet. Gynec.*, **69**, 606.
WILSON, T.—(1963) *J. Obstet. Gynaec. Br. Emp.*, **6**, 261.
WYNDER, E. L.—(1954) ' Cancer, Race and Geography '. Baltimore (Williams, Wilkins, Co.) pp. 25, 26, 129.—(1955) *Br. med. J.*, i, 743.
WYNDER, E. L., CORNFIELD, J., SCHROFF, P. D. AND DORAISWAMI, K. R.—(1954) *Am. J. Obstet. Gynec.*, **68**, 1016.